# Symptomatic aortitis at giant cell arteritis diagnosis: a prognostic factor of aortic event

**DOI:** 10.1186/s13075-020-02396-5

**Published:** 2021-01-07

**Authors:** Olivier Espitia, Gauthier Blonz, Geoffrey Urbanski, Cédric Landron, Jérôme Connault, Christian Lavigne, Pascal Roblot, François Maillot, Alexandra Audemard-Verger, Mathieu Artifoni, Cécile Durant, Béatrice Guyomarch, Mohamed Hamidou, Julie Magnant, Christian Agard, French Study Group for Large Vessel Vasculitis (GEFA)

**Affiliations:** 1 Department of Internal Medicine, CHU Nantes, 1 place Alexis Ricordeau, 44093 Nantes, France; 2 Department of Internal Medicine, CHU Angers, Angers, France; 3 Department of Internal Medicine, CHU Poitiers, Poitiers, France; 4 Department of Internal Medicine, CHRU Tours, Tours, France; 5 Research Department, Methodology and Biostatistics Platform, CHU Nantes, Nantes, France

**Keywords:** Giant cell arteritis, Aortitis, Aneurysm, Aortic dissection, Prognosis

## Abstract

**Background:**

Giant cell arteritis (GCA) is frequently associated with aortic involvement that is likely to cause life-threatening structural complications (aneurysm, dissection). Few studies have investigated the occurrence of these complications, and no predictive factor has been identified so far. The aim of this study was to investigate factors associated with the risk of aortic complications in a cohort of GCA aortitis.

**Methods:**

Data of all patients managed with aortitis (CT or 18 FDG PET) at the diagnosis of GCA in five hospitals from May 1998 and April 2019 were retrospectively collected. Clinical features were compared according to the presence of aortitis symptoms. The predictive factors of occurrence or aggravation of aortic structural abnormalities were investigated.

**Results:**

One hundred and seventy-one patients with GCA aortitis were included; 55 patients (32%) had symptoms of aortitis (dorsal/lumbar/abdominal pain, aortic insufficiency) at diagnosis. The median follow-up was 38 months. Aortic complications occurred after a median time of 32 months. There were 19 new aortic aneurysms or complications of aneurysm and 5 dissections. Survival without aortic complication was significantly different between the symptomatic and non-symptomatic groups (Log rank, *p* = 0.0003). In multivariate analysis the presence of aortitis symptoms at diagnosis (HR 6.64 [1.95, 22.6] *p* = 0.002) and GCA relapse (HR 3.62 [1.2, 10.9] *p* = 0.02) were factors associated with the occurrence of aortic complications.

**Conclusion:**

In this study, the presence of aortitis symptoms at the diagnosis of GCA aortitis and GCA relapse were independent predictive factors of occurrence of aortic complications during follow-up.

## Introduction

Giant cell arteritis (GCA) is the most frequent systemic vasculitis, in which aortitis is present at diagnosis in 40 to 65% of cases [1, 2]. The screening for aortitis is now consensually included in international guidelines for all patients diagnosed with GCA [3, 4]. Aortic involvement is not systematically evaluated, although it is a potentially life threatening condition due to aortic complications such as aortic aneurysms and dissection [5–9]. Aortic complications may be present at the time of diagnosis but may also occur many years after the diagnosis of GCA aortitis [9].

There are few data on the prognostic value of aortic involvement in GCA. Overall mortality does not appear to be increased [10, 11]. In contrast, in a cohort study of 204 GCA patients with large vessel involvement, survival was impaired in patients with aortic aneurysm or dissection [12]. Moreover, large vessel impairment was reported to be associated to higher GCA relapse rates [10, 11, 13, 14].

To date, there are no clear data on the link between clinical presentation of aortitis, at the time of diagnosis of GCA, and the prognosis of the disease. The aim of this study was to evaluate clinical presentation and outcome of GCA-related aortitis.

## Methods

### Patients

This study included patients diagnosed with GCA between May 1998 and April 2019 from 5 hospitals in western France. Data were collected from medical records. Each patient had to meet at least three American College of Rheumatology criteria for the diagnosis of GCA [15]; or be over 50-year-old age with a biological inflammatory syndrome and with the presence of an aortic inflammatory disease on imaging examination (CT, MRI, PET) [3]; and have an aortic involvement at diagnosis defined by the following: a circumferential aortic parietal thickening > 2.2 mm on CT/MRI, and/or a grade 2 or 3 parietal aortic hypermetabolism on PET [3, 16].

Symptomatic aortitis was defined by the presence 1 month before or at GCA diagnosis of one or more of the following signs: recent, less 2 months, occurrence of chest, dorsal, lumbar, or abdominal pain; or unknown aortic insufficiency with recent dyspnea highlighted at aortitis diagnosis. This signs were unexplained by any other cause than aortitis (musculoskeletal degenerative disease, atherosclerotic or other aortic disease).

### Ethics

This study have received ethics board approval by GNEDS (Groupe Nantais d’Ethique et de Soins) the local ethics committee of the University Hospital of Nantes (20200219). Each patient included in this study received written information, and no patient objected to this study.

### Definition of study end-points

Aortic complication was defined by the occurrence, at least 1 month after initial imaging of the aorta, of a new aortic structural abnormality (aneurysm: thoracic aortic diameter > 4 cm or abdominal aorta diameter > 3 cm; or aortic dissection), or the need for aortic surgery in response to a threatening structural abnormality (aortic dissection, progression of the aneurysm reaching a critical size, or aortic insufficiency).

Peripheral vascular event was defined by the occurrence, at least 1 month after initial imaging, of one of the following complication: limb ischemia, mesenteric or renal ischemia, myocardial ischemia, or ischemic stroke.

GCA relapse was defined by the concomitant reappearance of GCA-related clinical manifestations and a biological inflammatory syndrome (CRP ≥ 15 mg/l), or the reappearance of a biological inflammatory syndrome with inflammatory arterial parietal changes on CT, MRI or PET [17–20].

Patients with non-symptomatic aortitis (noS-Ao) at diagnosis were compared to patients with symptomatic aortitis at diagnosis (S-Ao).

Only radiographically monitored patients were included in the multivariate study and survival analysis.

### Statistical analysis

Qualitative values were expressed in terms of numbers and percentages. The mean comparisons were made using *t* test. Frequency comparisons were made by a chi-squared test or the Fisher test according to the statistical headcount. Prognostic factors associated with aortic complication were evaluated with Cox models. Hazard ratios (HR) with their 95%CI has been estimated as association measures. Variables with *p* < 0.05 in univariate model and all the variables already known to be confounding factors were candidate variables for multivariate model. Survival curves were estimated with their 95% confidence interval (95%CI) using Kaplan-Maier estimators, and Log rank tests were performed to compare aortic complication free survival between groups.

## Results

### Characteristics of the 171 patients with GCA aortitis

This study included 171 cases of GCA with aortitis at diagnosis, whose characteristics are described in Table 1. Aortic CT was performed at baseline in 142 patients (83%), both CT and PET in 67 (39%), PET in 92 (54%), and aortic MRI in 12 (7%). Ascending thoracic aorta was involved in 123 cases (72%), aortic arch in 107 (63%), descending thoracic aorta in 110 (64%), and abdominal aorta in 95 (56%). Thoracic aortic aneurysm at GCA diagnosis and abdominal aortic aneurysm was present respectively in 13 patients (23.6%) in S-Ao, 9 (7.8%) in noS-Ao (*p* = 0.009), 6 (10.9%) in S-Ao, and 4 (3.4%) in noS-Ao (*p* = 0.08).

**Table 1:** Population characteristics and comparison between symptomatic and non-symptomatic aortitis patients

	Total *n* = 171	noS-Ao *n* = 116	S-Ao *n* = 55	P
Characteristics at GCA diagnosis				
Gender (Female) n (%)	127 (74%)	91 (78%)	36 (65%)	0.1
Mean age at GCA diagnosis (years) ± SD	70 ± 9.0	70.6 ± 8.8	68.6 ± 9.5	0.19
Headache n (%)	86 (51%)	66 (57%)	20 (39%)	0.05
Temporal pulse abnormality n (%)	33 (21%)	27 (25%)	6 (13%)	0.14
Positive TAB n (%)	102 (60%)	73 (63%)	29 (53%)	0.27
CRP at diagnosis (mg/l) ± SD	104 ± 71.4	108 ± 74.6	68.6 ± 9.5	0.19
Mandibular impairment n (%)	31 (19%)	28 (25%)	3 (6%)	**< 0.01**
Fever n (%)	73 (43%)	57 (49%)	16 (3%)	0.17
Ophthalmological impairment n (%)	23 (14%)	19 (17%)	4 (7%)	0.16
Asthenia n (%)	139 (82%)	100 (87%)	39 (71%)	**0.02**
Weight loss n (%)	89 (53%)	65 (57%)	24 (44%)	0.19
PMR n (%)	37 (22%)	27 (24%)	10 (19%)	0.58
Aortic aneurysm n (%)	32 (19%)	13 (11%)	19 (35%)	**< 0.001**
Aortic dissection n (%)	8 (5%)	2 (2%)	6 (11%)	**0.01**
Aortic thickening in mm ± SD	4 ± 1.5	3.6 ± 1.0	4.6 ± 1.8	**0.001**
Cardiovascular risk factors				
Arterial hypertension n (%)	75 (44%)	44 (38%)	31 (56%)	**0.04**
Active or withdrawn smoking < 3 years n (%)	38 (22%)	20 (17%)	18 (33%)	**0.04**
Hypercholesterolemia n (%)	36 (21%)	22 (19%)	14 (25%)	0.46
Obesity (BMI > 30Kg/m^2^) n (%)	20 (12%)	11 (10%)	9 (17%)	0.3
Diabetes n (%)	15 (9%)	10 (9%)	5 (9%)	> 0.99
Treatment				
Initial prednisone dose (mg/kg/d) ± SD	0.8 ± 0.2	0.9 ± 0.2	0.8 ± 0.3	0.55
Perfusion of methylprednisolone at diagnosis, n (%)	28 (17%)	24 (21%)	4 (7%)	**0.046**
Immunosuppressive treatment introduced at diagnosis, n (%)	7 (4%)	6 (5.5%)	1 (2%)	0.43
Antiplatelet introduced at diagnosis, n (%)	107 (65%)	75 (66%)	32 (63%)	0.78
Statin treatment introduced at diagnosis, n (%)	36 (22%)	28 (25%)	8 (16%)	0.26
Follow-up				
Mean prednisone at 12 months (mg/d) ± SD	7.23 ± 5.6	7.83 ± 6.5	6.12 ± 3.3	0.08
Immunosuppressive treatment introduced during follow-up, n (%)	31 (18.1%)	24 (20.7%)	7 (12.7%)	0.23
Aortic imaging during follow up ≥2 evaluations n (%)	56/98 (57%)	27/54 (50%)	29/44 (66%)	0,11
Median time between aortitis diagnosis and last aortic evaluation (months) [min-max]	24 [1–180]	23.5 [1–180]	25 [4–109]	0.77
Median follow-up (months) [min-max]	38 [0–237]	30 [0–237]	55 [7–139]	0.41

*no-SAo* non-symptomatic aortitis, *S-Ao* symptomatic aortitis, *TAB* temporal artery biopsy, *CRP* C-reactive protein, *PMR* polymyalgia rheumatica, *BMI* body mass index

Aortitis was symptomatic in 55 cases (32%), including 51% chest pain, 31% abdominal pain, 16% back pain, and 35% had previously unknown aortic insufficiency with recent dyspnea.

Ten patients (5.8%) had undergone aortic surgery for an inaugural complication: 5 Stanford-A aortic dissections (1 patient died), and 5 ascending thoracic aorta aneurysms (all underwent surgery because of the size of the aneurysms, of which 3 were also responsible for severe aortic insufficiency).

Immunosuppressive treatment was introduced in 18.1% (*n* = 31) of cases (12.7% in S-Ao and 20.7% in noS-Ao (*p* = 0.20)): methotrexate in 23 cases, tocilizumab in 3 cases, azathioprine in 3 cases, and cyclophosphamide in 2 cases.

At least one control imaging (CT angiography, PET, or MR angiography) was performed for 98 patients: 44 (80%) of S-Ao patients and 54 (52%) of noS-Ao patients. For patients who had a control imaging during follow-up, mean duration between initial and last imaging was 35.6 months (± 34.7), mean follow-up time was 34.4 months (± 30.8) for S-Ao patients and 36.5 months (± 37.9) for noS-Ao patients (*p* = 0.77).

Twenty-three aortic complications occurred during follow-up (23.5% of patients with control imaging): 15 in S-Ao patients (34.1% with control imaging) and 8 in noS-Ao patients (14.8% with control imaging) (*p* < 0.01); these complications do not include aortic aneurysms, dissections or surgeries that occurred at diagnosis or in the month following the diagnosis of GCA. There were 15 new aortic aneurysms, 4 critical increase size of a pre-existing aneurysm requiring surgery, and 4 aortic dissections: 3 (5.5%) in S-Ao and 1 (0.9%) in noS-Ao (*p* = 0.06).

Aortic complications occurred after a median delay of 27 months [2 to 101 months]. One patient died following the rupture of an abdominal aortic aneurysm that was not present at diagnosis. These aortic complications required surgery for 9 patients, while 3 other had contraindications for surgery because of their health condition, and 1 declined surgery. Of the 15 aortic aneurysms that occurred during the follow-up, 11 were located on the ascending thoracic aorta, and 4 on the abdominal aorta.

### Vascular evolution and relapse in GCA aortitis patients

Multivariate cox model (Table 2) showed that the presence of aortitis symptoms at diagnosis (HR 6.64 [1.95, 22.6] *p* = 0.002) and GCA relapse (HR 3.62 [1.2, 10.9] *p* = 0.02) were independent factors of aortic complication.

**Table 2:** Univariate and multivariate Cox proportional hazards regression analyses of variables related to the occurrence of aortic complications on radiographically monitored patients (IS Immunosuppressive treatment)

Variable	Univariate analysis			Multivariate analysis		
	Hazard ratio	CI 95%	*p*	Hazard ratio	CI 95%	*p*
Age	1.02	0.96; 1.08	0.61	0.98	0.91; 1.04	0.42
CRP	0. 99	0.99; 1.00	0.54			
Positive TAB	0.79	0.31; 2.02	0.62			
Male gender	2.27	0.90; 5.77	0.08	1.39	0.41; 4.66	0.60
Headache	0.41	0.15; 1.13	0.09			
Mandibular symptoms	0.04	0.00; 132.24	0.44			
History of PMR prior to GCA diagnosis	2.00	0.58; 7.29	0.30			
Symptomatic aortitis	7.54	2.17; 26.23	0.001	**6.64**	**1.95; 22.58**	**0.002**
Tobacco	2.26	0.86; 5.95	0.10			
Hypertension	1.36	0.54; 3.44	0.51	2.50	0.92; 6.77	0.7
Hypercholesterolemia	1.73	0.65; 4.58	0.27			
Obesity	0.72	0.21; 2.41	0.59			
Diabetes	1.25	0.34; 4.63	0.74			
Aortic aneurysm at GCA diagnosis	3.12	1.20; 8.12	0.02	2.43	0.76; 7.77	0.14
Aortic dissection	0.49	0. 06; 3.75	0.49			
Aortic thickening > 3 mm	1.39	0.48; 4.05	0.54			
Perfusion of methylprednisolone at diagnosis	0.43	0.00; 119.63	0.44			
Prednisone 1 mg/kg/d at diagnosis	0.37	0.14; 0.99	0.05			
IS at GCA diagnosis	0.05	0.00; 580 e10^9^	0.84			
Antiplatelet at diagnosis	1.44	0.51; 4.04	0.49			
GCA relapse	1.31	0.48; 3.56	0.60	**3.62**	**1.21; 10.89**	**0.02**

**Fig. 1:**
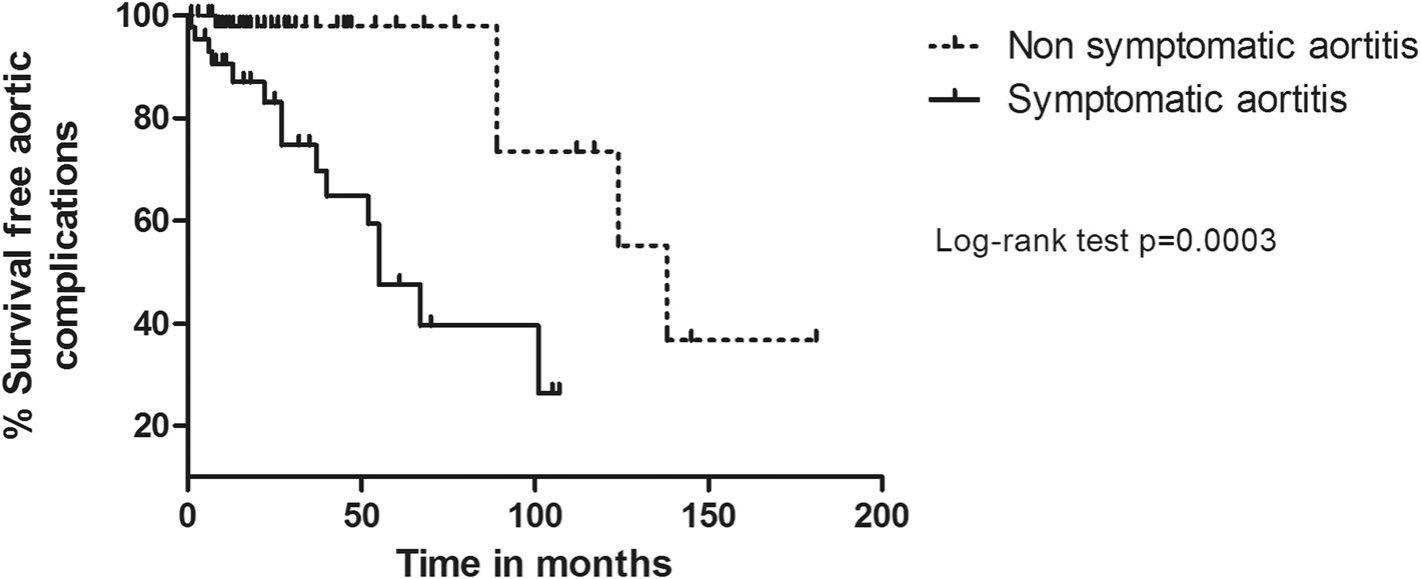
Survival without aortic complication: comparison according to the presence of aortitis symptoms at GCA diagnosis on radiographically monitored patients

Survival without aortic complication was significantly different between the symptomatic and non-symptomatic groups (Log rank, *p* = 0.0003) (Fig. 1).

Nineteen (35%) S-Ao and 53 (46%) noS-Ao patients had a GCA relapse (*p* = 0.18); 8 (15%) S-Ao and 21 (18%) noS-Ao patients developed subsequent peripheral vascular event (*p* = 0.72). Eighteen patients died during follow-up (2 S-Ao and 16 noS-Ao, *p* = 0.07). Causes of death recorded were 1 dissection of the thoracic aorta, 1 rupture of an abdominal aortic aneurysm, 5 cardiovascular causes, 3 infections, 1 cancer, and 1 severe hemorrhage.

## Discussion

This present study is the first aimed to assess the prognostic impact of symptomatic aortitis in a large cohort of GCA patients with well-documented aortitis at diagnosis.

Patients with symptomatic aortitis had significantly more cardiovascular risk factors (smoking, hypertension) than asymptomatic patients, suggesting a potential additional effect leading to severe aortic involvement. In accordance to the literature, the aorta was mostly affected in its thoracic segment, especially the ascending thoracic aorta [1, 2]. Aortic aneurysms may be present at the time of diagnosis of GCA, concerning between 4 and 23% of patients [1, 2]. In our present study, exclusively including patients with well-documented aortitis, this frequency was quite high (19%). At diagnosis or during follow-up, de Boysson et al. reported the discovery of aortic aneurysms in 11% of patients with aortitis, but also in 7% of GCA patients without aortitis [11]. In this study, an aortic aneurysm or dissection was inaugural in 23.4% of patients, with a need for surgery at diagnosis in 5.8% of all patients. These data support the interest of performing aorta imaging at diagnosis of GCA [4].

In our experience, patients with non-symptomatic aortitis received more frequently Methylprednisolone pulses. This is in part due to a higher frequency of ocular involvement in these patients. The use of immunosuppressive treatment during follow-up remained infrequent (18.1% of all cases) and mainly used for severe or resistant forms.

In case of aortitis, control imaging should be used to assess evolution and detect structural complications. The lack of systematic monitoring probably leads to an underestimation of the actual number of aortic complications. In the presence of aortitis, imaging at 1 and 2 years from the diagnosis may be considered to assess wall inflammation and screen for aortic aneurysms; later imaging can be discussed in case of multiple relapses or corticosteroid dependence.

During GCA, possible predictive factors of aortic complication (aneurysm, dissection) have already been suggested: coronary artery disease and hypercholesterolemia [7, 12], arterial hypertension and PMR at diagnosis [21], and presence of aneurysms of the subclavian arteries [22], but prospective studies are lacking to assess this important issue. In a study including 549 patients, aortic inflammation has been described as the best predictor of aortic dilation [11]. In our own previous study, aortitis at the diagnosis of GCA was associated to higher corticosteroid dependence and higher cardiovascular mortality [8].

In this study, 23.5% of patients (14.8% of noS-Ao patients and 34.1% of S-Ao patients) developed an aortic complication after a median delay of 27 months, which is quite similar to the 2.5 years found in the historical study by Evans et al. [5]. Moreover, we found that symptomatic aortitis at diagnosis was associated to a significantly higher risk of developing aortic complications, with hazard ratio at 6.64. The presence of aortitis symptoms could therefore reflect a disease with locally more severe inflammation that is the basis for structural damage. The interpretation of the prognosis of symptomatic aortitis must be careful because it also can be argued that advanced aortic disease at GCA diagnosis has unfavorable prognosis due to a significant diagnostic delay or a prior aortic disease.

This study presents an original concept with the search for clinical signs of aortitis at GCA diagnosis. If these results are confirmed, imaging could help identify patients with more aggressive aortitis.

Our study has several limitations: with the potential data capture errors and missing data that are inherently an issue with all retrospective data collection and short median follow-up. Although the initial dose of corticosteroid therapy was the same in both groups, tapering schedules were not standardized, and the use of immunosuppressive therapy was not standardized. Moreover, for most patients, control modalities and blood pressure objectives were not clearly indicated. Only 18.1% of the cohort were treated with additional immunosuppression despite recent EULAR recommendations [23] since this is a cohort with patients included over a long period with some patients exclusively managed with steroids. The follow-up time limited the ability to capture aortic complication that can occur 5–10 years after diagnosis.

In addition, clinical signs associated with symptomatic aortitis are non-specific: pain or dyspnea is very frequent in elderly population; however in this study, symptomatic aortitis was retained after exclusion of musculoskeletal degenerative disease and atherosclerotic or other aortic disease that could explain these symptoms.

Several aortic CT scans performed were not synchronized to the heart rate (limiting the analysis of the ascending thoracic aorta) and some had no late arterial phase to evaluate aortic parietal contrast; follow-up imaging was not systematic and was performed at widely varying times.

If these data are confirmed in prospective studies, a more intensive initial treatment for symptomatic aortitis at diagnosis—with initial corticosteroid therapy combined with immunosuppressive therapy—could be evaluated.

The management of cardiovascular risk factors is also fundamental, including blood pressure monitoring with self-measures, a strict goal for LDL cholesterol level, smoking cessation, and regular physical activity.

## Conclusion

Aortitis in GCA may lead to severe complications like aneurysm or dissection. Patients who have symptomatic aortitis, mainly chest and abdominal pain, at the time of diagnosis of GCA, could represent a distinct sub-group of aortitis with more aortic aneurysm or dissection during follow-up than patients with non-symptomatic aortitis. Prospective studies are needed to confirm our results. In order to take better care of these patients, recommendations on the frequency and modalities of aortic imaging remain to be defined.

## Data Availability

Data are available to request. No expiration date of data requests is currently set once they are made available. Access is provided after a proposal has been approved by an independent review committee identified for this purpose and after receipt of a signed data sharing agreement. Data and documents, including the study protocol, statistical analysis plan, and clinical study report, will be provided in a secure data sharing environment for up to 2 years per proposal. For details on submitting a request, see the instructions provided at www.clinicalstudydatarequest.com.

## Abbreviations

CT: Computed tomography
GCA: Giant cell arteritis
noS-Ao: Non-symptomatic aortitis
PET: Positron emission tomography
S-Ao: Symptomatic aortitis
